# FTY720 Reduces Endothelial Cell Apoptosis and Remodels Neurovascular Unit after Experimental Traumatic Brain Injury

**DOI:** 10.7150/ijms.49066

**Published:** 2021-01-01

**Authors:** Hao Cheng, Guangfu Di, Chao-Chao Gao, Guoyuan He, Xue Wang, Yan-Ling Han, Le-an Sun, Meng-Liang Zhou, Xiaochun Jiang

**Affiliations:** 1 Department of Neurosurgery, Yijishan Hospital, Wannan Medical College, Anhui, China.; 2 Department of Neurosurgery, Jinling Hospital, Jinling School of Clinical Medicine, Nanjing Medical University, Jiangsu, China.; 3 Department of Neurosurgery, Jinling Hospital, School of Medicine, Nanjing University, Jiangsu, China.

**Keywords:** FTY720, sphingosine-1-phosphate receptor 1, traumatic brain injury, blood-brain barrier, endothelial cell

## Abstract

Traumatic brain injury (TBI) is a major cause of death and disability worldwide. A sequence of pathological processes occurred when there is TBI. Previous studies showed that sphingosine-1-phosphate receptor 1 (S1PR1) played a critical role in inflammatory response in the brain after TBI. Thus, the present study was designed to evaluate the effects of the S1PR1 modulator FTY720 on neurovascular unit (NVU) after experimental TBI in mice. The weight-drop TBI method was used to induce TBI. Western blot (WB) was performed to determine the levels of SIPR1, claudin-5 and occludin at different time points. FTY720 was intraperitoneally administered to mice after TBI was induced. The terminal deoxynucleotidyl transferase-dUTP nick end labeling (TUNEL) assay was used to assess endothelial cell apoptosis. Immunofluorescence and WB were performed to measure the expression of tight junction proteins: claudin-5 and occludin. Evans blue (EB) permeability assay and brain water content were applied to evaluate the blood-brain barrier (BBB) permeability and brain edema. Immunohistochemistry was performed to assess the activation of astrocytes and microglia. The results showed that FTY720 administration reduced endothelial cell apoptosis and improved BBB permeability. FTY720 also attenuated astrocytes and microglia activation. Furthermore, treatment with FTY720 not only improved neurological function, but also increased the survival rate of mice significantly. These findings suggest that FTY720 administration restored the structure of the NVU after experimental TBI by decreasing endothelial cell apoptosis and attenuating the activation of astrocytes. Moreover, FTY720 might reduce inflammation in the brain by reducing the activation of microglia in TBI mice.

## Introduction

Traumatic brain injury (TBI) is one of the major health problems worldwide, which is a prominent cause of death and long-term disability in young adults 1. TBI can be divided into primary and secondary injuries 2. Primary TBI is defined as the direct physical injury to the brain such as compression. Subsequently, a cascade of inflammatory, metabolic and biochemical changes are initiated, which leads to the secondary brain injury 3. Molecular and cellular responses occurred in the injured brain after TBI induce brain edema, hemorrhage and ischemia 4, 5, which are dynamic conditions that continue to change after onset 3. Given this complexity, there is an urgent need to better understand the pathophysiological mechanisms of TBI, especially the mechanisms of the secondary brain injury.

**Figure 1:**
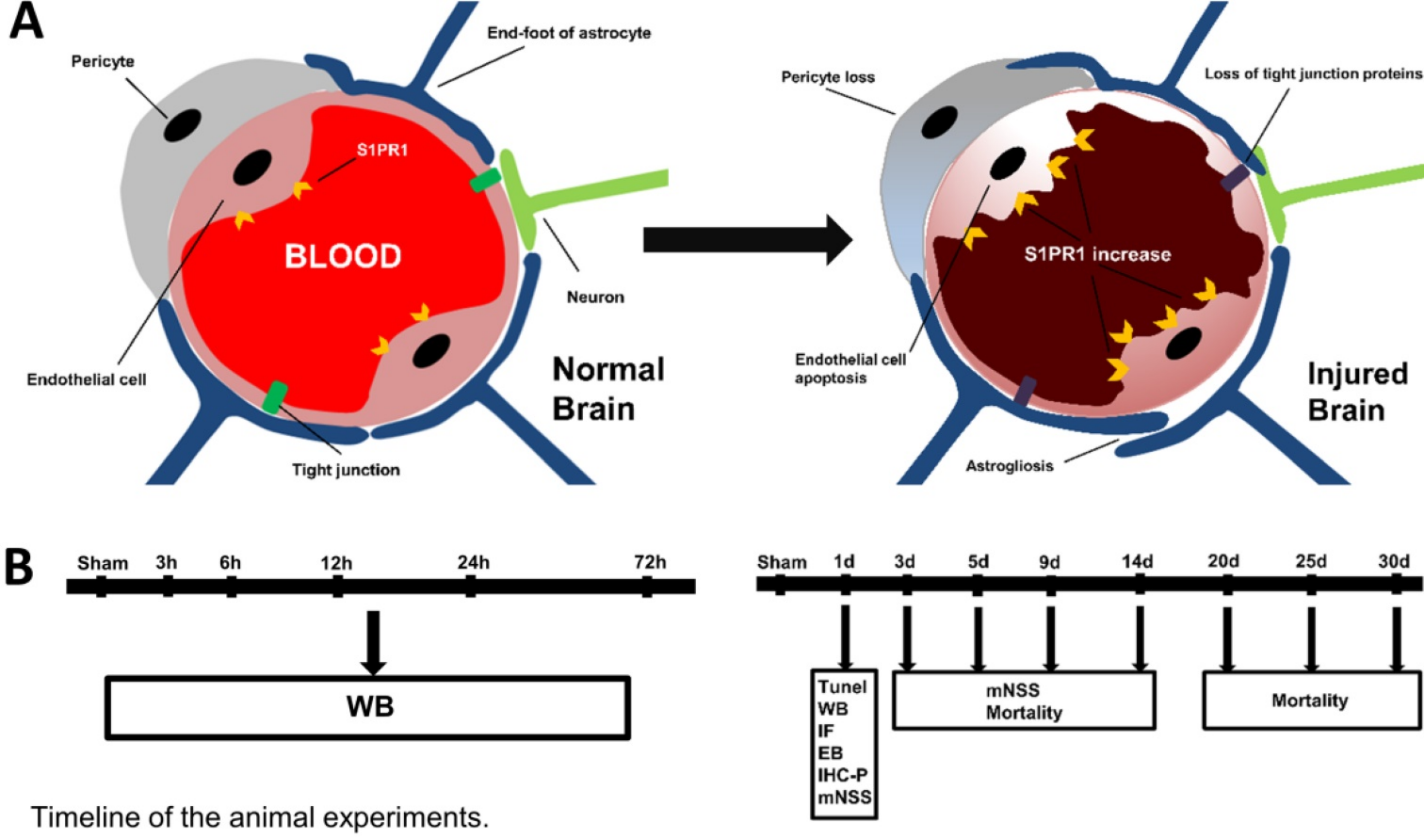
** A.** The structure of the neurovascular unit (NVU) in normal and injured brain. The NVU consists of endothelial cells, perivascular astrocytes, microglia, and neurons. After injury, the structure of NVU is severely disturbed, which includes the decreases of tight junction proteins, astrogliosis, pericyte loss, endothelial cell apoptosis. **B.** The timeline of animal experiments in the present study.

The normal function of the brain depends on a steady circulation of blood in the network of blood vessels including arteries, arterioles, capillaries venules and veins. Neurovascular unit (NVU) constituted by the brain parenchyma and vascular network, is essential for the health and function of the central nervous system (CNS) 6. As indicated in Fig. 1, NVU is mainly composed of blood-brain barrier (BBB) and perivascular neural cells including astrocytes, microglia and neurons. Endothelial cells, as the anatomical substrate of the BBB, have great significance for the structural integrity of BBB and NVU 6, 7. Once endothelial cells were damaged by TBI, it would lead to BBB destruction, and thus triggered some corresponding dysfunction 8, 9. The other component of the NVU are astrocytes, the most widely distributed type of cells in the brain, which extend and fill between the cell body and the processes or fibers of neurons. They play the role of supporting and separating different kinds of cells in CNS 10. Besides, the processes of astrocytes often expand to form end-feet, which attach to adjacent endothelial cells and participate in the formation of NVU 4.

Microglias are the main immune cells in the brain and play a role in brain homeostasis and various neurological disorders. Numerous studies have shown that activated microglia play an important role in CNS diseases such as TBI, Parkinson's disease and Alzheimer's disease. Nonetheless, excessive activated microglia would cause neurotoxicity by acting as an important source of pro-inflammatory factors and oxidative stress, such as tumor necrosis factor (TNF), nitric oxide, interleukin and other neurotoxic substances 11.

Sphingolipids are important signaling molecules involved in many pathophysiological processes. Sphingosine-1-phosphate (S1P) is a kind of metabolite of sphingolipids, which are involved in many physiological reactions such as cell differentiation, apoptosis and proliferation 12. S1P receptors (S1PRs), a series of G-protein-coupled receptors, are widely distributed on the cell surface, which were demonstrated to be expressed in the CNS 1, 13. S1PR1 is mainly expressed in endothelial cells, macrophages and cardiomyocytes, which play an important role in regulating angiogenesis, inflammatory cell infiltration and immune response 14, 15. A recent study demonstrated that endothelial-specific S1PR1 knockout mice (S1pr1iECKO) showed BBB breach for small-molecular-mass fluorescence tracers 16. FTY720 is an oral S1PR1 functional modulator extracted from cordyceps sinensis, which can be combined with S1PR1 to play an critical role in immunomodulatory 17, 18. In the present study, we investigated the effects of S1PR1 agonist FTY720 on the permeability of BBB and the activation of astrocytes and microglia after TBI.

## Materials and methods

### Animal ethical approval

ICR mice (purchased from Qinglongshan Animal Center, Nanjing, China) were used at 6-8 weeks of age; weighing 28-32 g. Mice were housed in individual cages and fed under a 12/12-hour light/dark cycle at 22 ± 2 °C and 50 ± 5% humidity. Regular laboratory diet and tap water were available *ad libitum*. All experiments and protocols were abided by the Guide for the Care and Use of Laboratory Animals by the National Institutes of Health (NIH) and approved by the Animal Care and Use Committee of Jinling Hospital.

### TBI model

TBI was induced in mice by a weight-drop method, as previously described 19. In brief, mice were anesthetized with isoflurane, and were then placed on the platform under the weight-drop device. In order to expose the skull, a 1.5 cm midline longitudinal scalp incision was made. The left anterior frontal area (the exact location is 1.5 mm lateral to the midline on the mid-coronal plane) was used as the impact area, a 200 g weight was released above the impact area from a height of 2.5 cm. Then, the skin incision was sutured immediately, and the mice were returned to their own cages.

### Experimental groups

Initially, mice were randomly allocated into six groups (n=6 for each group): sham group (mice were subjected to scalp incision procedure only) and TBI groups (3, 6, 12, 24 and 72 hours [h]). Sham mice were sacrificed right after operation and TBI mice were euthanized at the indicated time-point after TBI. Their brain samples were collected for further analysis.

Furthermore, mice were randomly assigned to 4 groups: 1) sham group, 2) TBI group, 3) TBI + FTY720 group: FTY720 (0.5 mg/kg, Selleck chemicals, Houston, TX, USA) was intraperitoneally administered right after TBI. 4) TBI + vehicle group, equal volumes of vehicle [1% dimethylsulfoxide (DMSO)] were injected immediately after TBI. FTY720 with a purity of more than 99% was dissolved in saline containing 1% DMSO.

### Tissue preparation

Animals were anesthetized with isoflurane and were perfused intracardially with cold (4 °C) heparinized 0.9% saline. For Western blot analysis, brain tissues around the injured site were collected. Samples were immediately frozen in liquid nitrogen, then transferred to a -80 °C freezer until use. For frozen and paraffin sections, the brain was steeped in formaldehyde solution after perfusion.

### Assay for S1P levels

ELISA tests were performed using a commercial kit (Echelon Biosciences, UT, USA). The grouping is shown above; S1P quantitative analysis was performed on all grouped brain tissue homogenate samples in duplicate. The levels were expressed as pmol/mg of sphingolipid.

### Modified neurological severity score

To evaluate the neurological impairments of mice after TBI, we used the modified neurological severity score (mNSS), which include motor, sensory, balance beam and reflex tests. One point is given for failing to perform each of the tasks. The score of mNSS ranged from 0 (normal function) to 18 (maximal deficit). A higher score indicated severer impairment of neurological function. In the present study, mNSS were assessed on days 1, 3, 5, 9, and 14 days post-TBI. All neurobehavioral tests were carried out by two investigators who were blinded to the experimental groups.

### Brain water content

Brain water content was measured by the dry-wet weight method. Mice were sacrificed 24 h post-TBI. After anesthesia, the brains were removed immediately and divided into ipsilateral hemispheres (with injury) and contralateral hemispheres. The ipsilateral hemispheres were weighed immediately to obtain the wet weight and then dried at 80 °C for 72 h to obtain the dry weight. As previously described 20, the percentage of water content was calculated as follows: brain water content = [(wet weight - dry weight)/wet weight] × 100%.

### Evans blue permeability assay

In order to evaluate BBB permeability, mice were injected with 2% Evans blue (EB, 3 ml/kg) (Sigma-Aldrich, St. Louis, MO, USA) *via* the tail vein at 24 h after TBI. One hour later, the mice were anesthetized with isoflurane and then perfused with phosphate-buffered saline (PBS) to remove the intravascular EB dye. The brains were obtained for imaging and EB content measurement. Briefly, the ipsilateral hemispheres were weighted and homogenized in 0.4 ml of formamide. The samples were incubated in a 37 °C water bath for 24 h. Then, samples were centrifuged at 10000 g at 4 °C for 10 min. The supernatant was collected and EB absorbance was measured by absorbance spectroscopy at 610 nm.

### Western blot analysis

Samples containing equivalent amounts of protein were separated using sodium dodecyl sulfate-polyacrylamide gel electrophoresis (SDS-PAGE) (7.5%-10%) and then were transferred to polyvinylidene difluoride (PVDF) membranes. The membranes were blocked for 2 h in a blocking buffer [Tris-buffered saline with 0.05% Tween 20 (TBST) containing 5% skim milk] and then incubated overnight at 4 °C with primary antibodies (claudin-5, 1:1000, Invitrogen, Carlsbad, CA, USA; occludin, 1:1000, Abcam, Cambridge, MA, USA; S1PR1, 1:1000, Abcam, Cambridge, MA, USA; p-extracellular regulated protein kinases (ERK) 1/2, 1:500, Santa Cruz Biotechnology, Santa Cruz, CA, USA; ERK 1/2, 1:500, Santa Cruz Biotechnology, Santa Cruz, CA, USA). After incubation with corresponding secondary antibodies at room temperature for 1 h, the protein bands were estimated by Immobilon Western Chemiluminescent horseradish peroxidase substrate. All protein bands were visualized by enhanced chemiluminutesescence (ECL) kit (EMD Millipore, Billerica, MA, USA) and were recorded by Tanon 5500 Chemiluminescence Imaging system (Tanon Science and Technology Co., Ltd., Shanghai, China). ImageJ software (US National Institutes of Health) was used to analyze the blots.

### Terminal deoxynucleotidyl transferase-mediated dUTP nick 3'-end labeling (TUNEL) staining and immunofluorescence staining of CD31

TUNEL staining was performed to detect dying cells using an In Situ Cell Detection Kit (Roche, Nutley, NJ, USA) according to the manufacturer's instructions. TUNEL and immunofluorescence staining of CD31 were combined to assess endothelial cell apoptosis. Briefly, paraffin sections were incubated with anti-CD31 antibody (1:500, Abcam, Cambridge, MA, USA) at 4 °C for 24 h. After 1 h incubation with Cy3-conjugated anti-mouse IgG (1:100, Thermo Fisher Scientific, Waltham, MA, USA), 50 µl of TUNEL mixture was added. Then the sections were continued to incubate at 37 °C for 1 h. At the end, 4',6-diamidino2-phenylindole (Dapi, 1:50000, Kaiji Biological, Nanjing, China) was added and incubated for 4 min. A fluorescence microscope was used to determine the number of apoptotic endothelial cells around the contusion area.

### Immunofluorescence staining of occludin and claudin-5

The procedures for immunofluorescence staining of occludin and claudin-5 were similar with that for CD31 as described above. The sections were incubated with primary antibodies of occludin (1:200) or claudin-5 (1:200). Dapi was also used to counterstain. The sections were observed under a fluorescence microscope.

### Immunohistochemistry

Brain sections were incubated with 3% H_2_O_2_ for 15 min to block endogenous peroxidases, then washed three times with PBS and incubated for 1 h at room temperature with a blocking solution (10% goat serum). Subsequently, sections were incubated overnight with primary antibodies (GFAP, 1:500, Abcam, Cambridge, MA, USA; IBA-1, 1:200, Abcam, Cambridge, MA, USA). Then the sections were washed with PBS followed by incubation for 1 h at room temperature with biotinylated secondary antibodies. After washing with PBS again, sections were incubated for 30 min with avidin/biotinylated horseradish peroxidase. Finally, sections were washed with PBS and reacted with DAB as a chromogen.

### Statistical analysis

SPSS statistical software (version 24.0, IBM) was used for all statistical analyses in the present study. The results are presented as the mean ± SD and were analyzed using a one-way analysis of variance (ANOVA) with least significant difference (LSD) or Tamhane post hoc test. Survival curves were generated and compared with the log rank test. A *p*-value < 0.05 was statistically significant.

## Results

### Time-course expressions of S1P, SIPR1, claudin-5 and occludin after TBI

**Figure 2:**
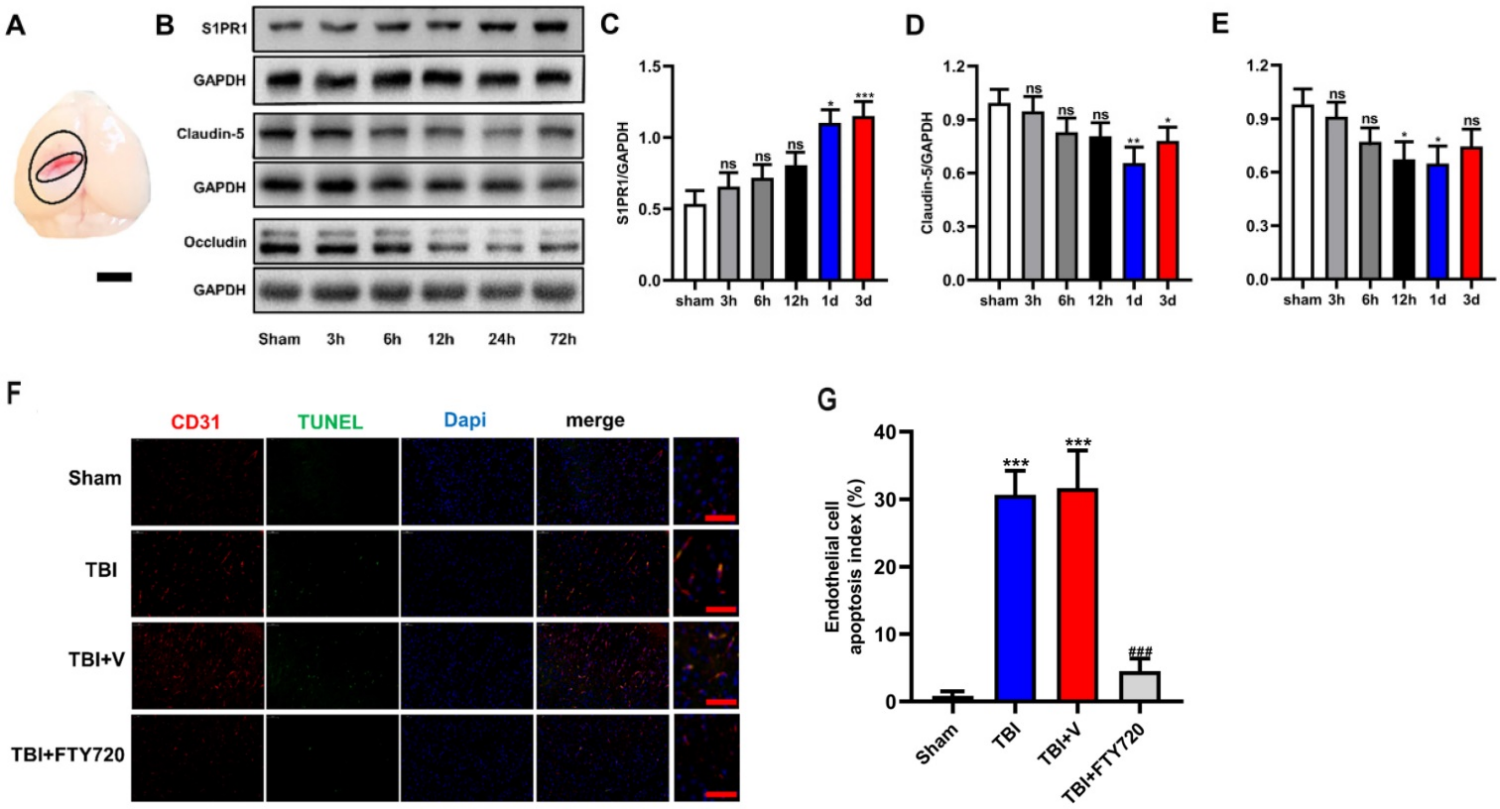
** A.** Brain samples for Western blot analysis were taken as illustrated. The tissues from the penumbra area surrounding the trauma tissue was collected. **B-E.** The protein levels of S1PR1, claudin-5 and occludin in the sham group and in TBI groups (3, 6, 12, 24 and 72 h) were detected by Western blot analysis. Then, the intensity of the blots was quantified and analyzed by image J. Data were represented as the means ± SD (n = 6 per group), *p < 0.05, **p < 0.01, ***p < 0.001 versus the sham group. **F.** FTY720 treatment decreased the endothelial cell apoptosis. Fluorescence colors: CD31 (red); TUNEL(green); Dapi(blue). Scale bar, 50 µm. CD31, TUNEL and Dapi triple stained cells represented the apoptotic endothelial cells. **G.** Quantification of apoptotic endothelial cells in different groups. Data are presented as mean ± SD (n = 3 per group), *p < 0.05, **p < 0.01, ***p < 0.001 versus the sham group; #*p* < 0.05, ##*p* < 0.01, ###*p* < 0.001 versus the TBI +vehicle group. Scale bar, 50 µm.

We used Western blot analysis to detect the expressions of the molecules of tight junction. Samples around the lesion (Fig. 2A) were collected at different time-points after TBI. As shown in Fig. 2B, D, E, both occludin and claudin-5 reached the minimum at the time-point of 24 h (*p* < 0.05 for occludin and *p* < 0.01 for claudin-5). The results indicated that the expressions of tight junction proteins began to recover after 24 h. Therefore, we chose 24 h as the time node for the following experiments. And these findings imply the breakdown of BBB after TBI resulting from the reduction of tight junction proteins.

In addition, the expression of S1PR1 was also detected by Western blot analysis at different time-points to demonstrate the changes of S1PR1 after TBI. The results showed significant upregulation of S1PR1 after 24 h post-TBI when compared with the sham group (*p* < 0.05) (Fig. 2B, C). And the levels of S1P also changed after TBI, the concentration of S1P in TBI brain tissue at various time points were measured by Elisa. We found the concentration of S1P increased significantly from 6 hours (*p* < 0.05) (Supplementary fig. A).

### FTY720 administration reduced endothelial cell apoptosis caused by TBI

TUNEL assay was used to detect endothelial cell apoptosis. As showed in Fig. 2F, with immunofluorescence staining of CD31, a widely used marker of endothelial cells, the proportion of apoptotic endothelial cells in TBI and TBI + vehicle groups could be determined, which were significantly higher than that in TBI + FTY720 group (*p* < 0.001). These results suggested that FTY720 treatment can reduce the apoptosis of endothelial cells after TBI.

### FTY720 enhanced the expressions of tight junction proteins and decreased the expression of S1PR1 after TBI

**Figure 3:**
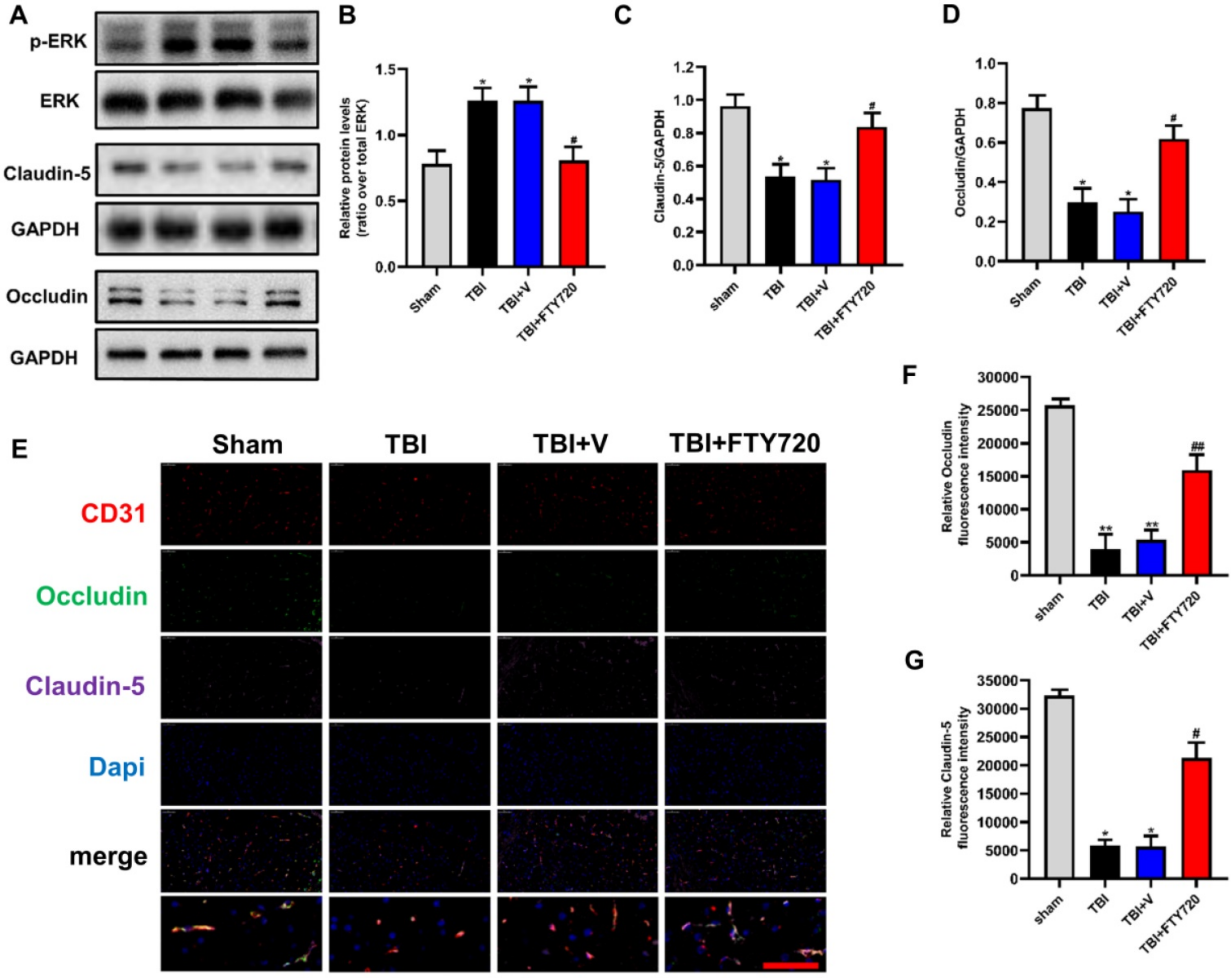
** A-D.** Protein levels of p-ERK, claudin-5 and occludin were measured by Western blot analysis (n = 6 per group). **E-G.** Changes and location of claudin-5 and occludin were also detected by immunofluorescence staining. Data are presented as mean ± SD (n = 3 per group), *p < 0.05, **p < 0.01, ***p < 0.001 versus the sham group; #*p* < 0.05, ##*p* < 0.01, ###*p* < 0.001 versus the TBI+ vehicle group. Scale bar, 50 µm.

In order to investigate the effects of FTY720 on the tight junction proteins after TBI, we analyzed the tight junction proteins by Western blot analysis and immunofluorescence. Compared with the sham group, the expressions of occludin and claudin-5 in the TBI group and the TBI + vehicle group were significantly decreased (*p* < 0.05), and both of them were significantly increased after FTY720 treatment (Fig. 3A, C, D). Immunofluorescent staining also showed that occludin and claudin-5 were down-regulated after TBI significantly and which were restored by FTY720 (Fig. 3E, F, G). Besides, activated (phosphorylated) ERK1/2, a key protein that transmits signals from surface receptors to the nucleus, was significantly elevated in the brain at 24 h after TBI. And this increase was reduced after treatment with FTY720 (*p* < 0.05) (Fig. 3A, B). Then we tested the expression of the target of FTY720, and our result found that the expression of S1PR1 decreased significantly after using FTY720 (*p* < 0.05) (Supplementary Fig. C, D). But FTY720 had no effect on the content of S1P after TBI (*p* > 0.05) (Supplementary Fig. B).

### FTY720 improved EB permeability and attenuated brain water content after TBI

**Figure 4:**
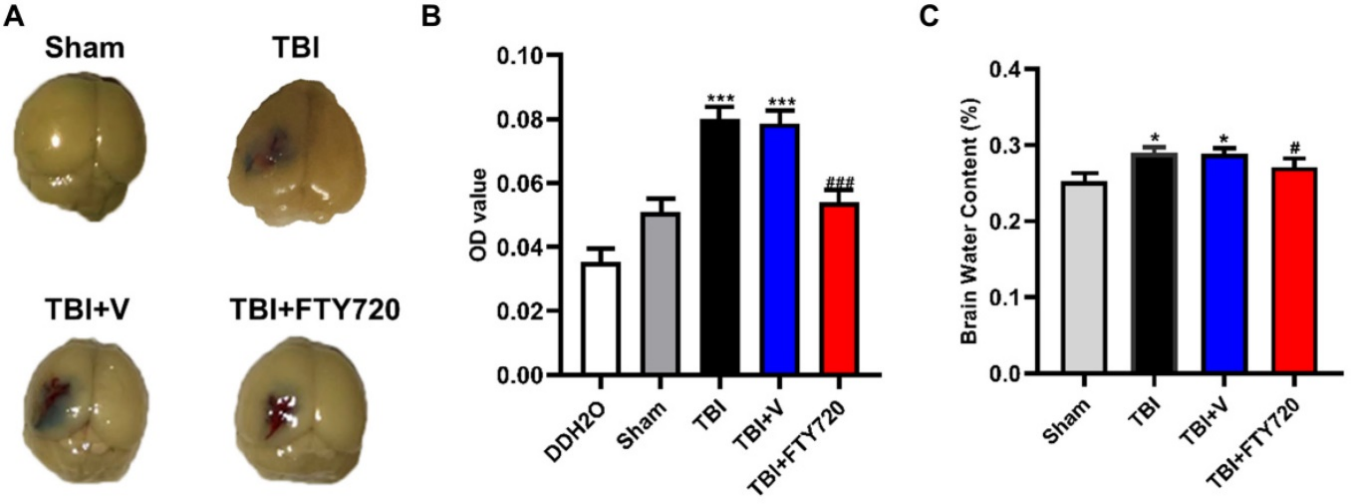
** A.** The mice were perfused after EB injection. The brain tissue stained with EB could be found in the area near the contusion. **B.** Photometric analysis of EB dye content showed that the EB content was higher in the TBI and TBI + vehicle groups than that in the TBI + FTY720 group. **C.** The animals in the TBI and TBI + vehicle groups had higher brain water content than the sham group. With administration of FTY720, brain water content was decreased. Data are presented as mean ± SD (n = 6 per group), *p < 0.05, **p < 0.01, ***p < 0.001 versus the sham group; #*p* < 0.05, ##*p* < 0.01, ###*p* < 0.001 versus the TBI + vehicle group.

The tight junction between endothelial cells is the structural foundation of the BBB. As mentioned above, increased endothelial cell apoptosis and decreased tight junction proteins could be found after TBI. EB permeability assay was used to detect the breakdown of BBB. EB permeability was significantly increased in the TBI and TBI + vehicle group compared with the sham group (*p* < 0.001), while the EB permeability was significantly reduced in the TBI + FTY720 group compared with the TBI + vehicle group (*p* < 0.001). These findings indicated that BBB breakdown resulting from TBI was relieved by FTY720 (Fig. 4A, B).

Additionally, brain water content was also assessed in the present study. As shown in Fig. 4C, the water content of brain tissue was higher in the TBI and TBI + vehicle group compared with the sham group (*p* < 0.05). Furthermore, the brain water content was reduced by FTY720 (*p* < 0.05). These results indicated that FTY720 can reduce brain edema caused by TBI.

### FTY720 attenuated astrocytes and microglia activation in TBI mice

**Figure 5:**
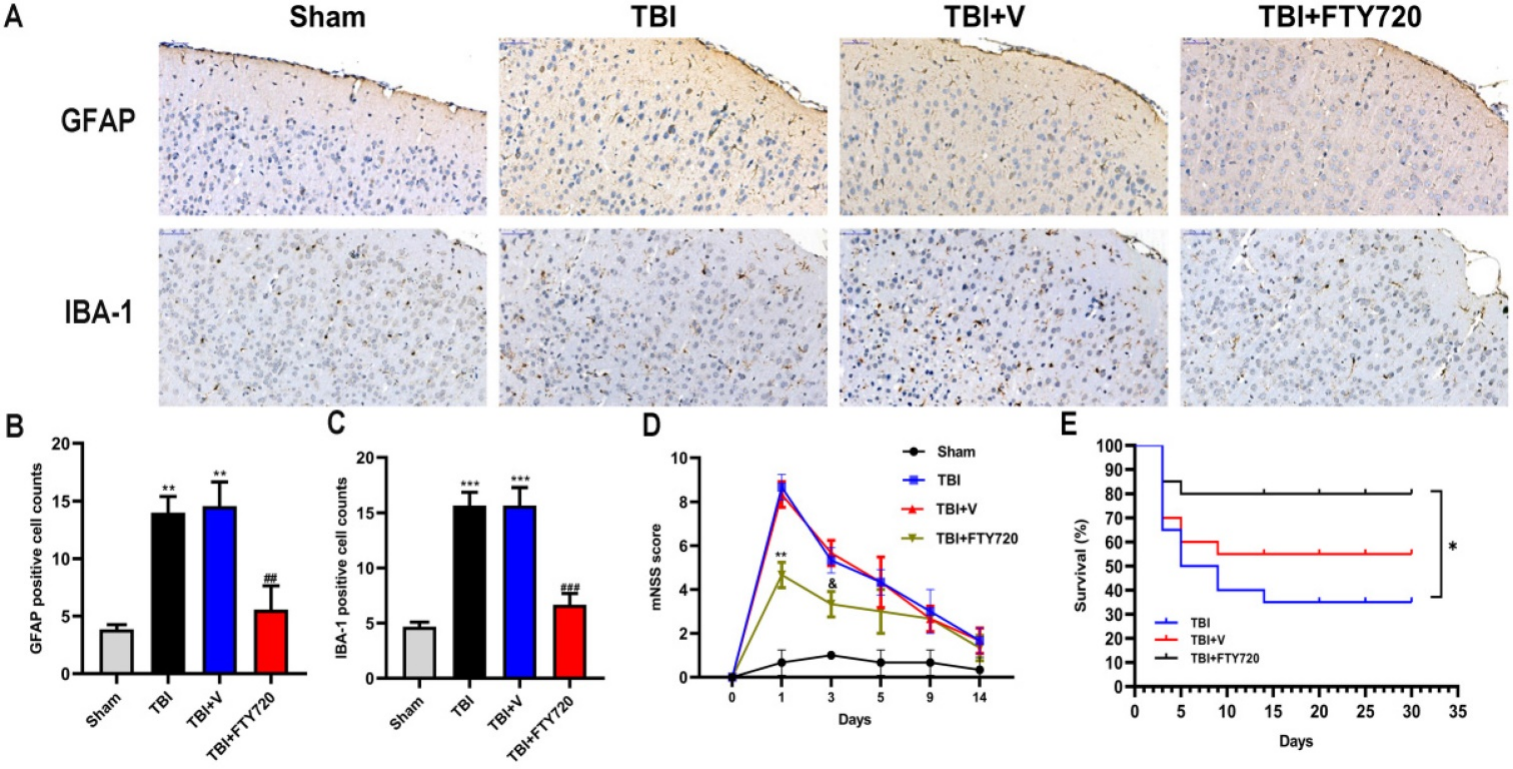
** A.** Altered astrocytic and microglial morphology. GFAP and IBA-1 positive cells were found by immunohistochemistry in four groups. **B,C.** Quantification analyses. Cell densities for GFAP and IBA-1 were shown. Data are presented as mean ± SD (n = 3 per group), *p < 0.05, **p < 0.01, ***p < 0.001 versus the sham group; #*p* < 0.05, ##*p* < 0.01, ###p < 0.001 versus TBI + vehicle group. Scale bar, 50 µm. **E.** The impact of FTY720 on the neurological function. The neurological function was evaluated with mNSS, n = 6/group. Data are presented as mean ± SD, *p < 0.05, **p < 0.01, ***p < 0.001 versus TBI + vehicle group; and *p* > 0.05 versus TBI + vehicle group. **F.** Survival analysis for each group, n = 20/group. At the end of the observation period, the mortality was 20% in the FTY720 group, 65% in the TBI group and 45% in the vehicle group. Data are presented as mean ± SD, *p < 0.05, **p < 0.01, ***p < 0.001 versus TBI + vehicle group; and *p* > 0.05 versus TBI + vehicle group.

To better understand the status of NVU after TBI, we evaluated astrocytes and microglia by immunohistochemistry. Astrocytes can form glial scar through proliferation when the CNS suffered from trauma, whose representative feature is the activation of astrocytes. As indicated in Fig. 5A, B, more activated astrocytes were detected around the injured tissue. After administration of FTY720, activated astrocytes were reduced (*p* < 0.001).

We also labeled the microglia/macrophages by immunohistochemistry. We can see activated microglia or macrophages labeled with IBA-1 around the damaged brain tissue. Similarly, FTY720 reduced activated microglia or macrophages surrounding the injured area (*p* < 0.001) (Fig. 5A, C). Together, these results demonstrated the ability of FTY720 to reduce the activations of astrocytes and microglia after TBI.

### FTY720 improved the neurological deficit and lower the mortality after TBI

As depicted in Fig. 5D, all the groups except the sham group presented higher mNSS scores at 24 h. But the score in the FTY720 treatment group was significantly lower than that in the TBI or TBI + vehicle group (*p* < 0.01). The neurological function of the mice in the TBI + FTY720 group was tended to be recovered at 72 h after TBI. However, no significant differences were found between groups. Moreover, we recorded the survival of the mice in 3 groups (TBI group, TBI + FTY720 group and TBI + vehicle group) (Fig. 5E). Survival analysis showed that deaths of mice started within 5 days in all groups. After 30 days of observation, 16 mice were alive in the FTY720-treated group (mortality, 20%), but only 7 mice were alive in the TBI group (mortality, 65%) and 11 mice left in the TBI + vehicle group (mortality, 45%) (*p* < 0.05).

## Discussion

Clinical evidence shows the dysfunction of brain microvascular accompanies the whole process of TBI 21. After TBI, the arterioles and capillaries in the cerebral cortex are extensively damaged, leading to the breakdown of the BBB 22. Hawkins BT et al. discovered that 1) endothelial intercellular connections were enlarged (as shown in Fig. 1); 2) tight junction proteins and pericytes were lost; 3) astrocytes were activated after TBI 22, 23. Occludin is a transmembrane connective protein that widely exists in endothelial cells and constitutes a tight intercellular linkage 6. Claudin-5 is also a tight junction protein that plays an important role in transporting small molecules through the BBB. We examined the changes of these two tight junction proteins in TBI mice at different time-points. Our results showed that occludin and claudin-5 decreased after TBI and reached the lowest levels at 24 h post-TBI. We speculated that the apoptosis of endothelial cells caused the loss of tight junction proteins. Our data confirmed this hypothesis with TUNEL assay and CD31 staining.

Finding drugs targeting the secondary brain injury is a great challenge to the researchers on TBI. As mentioned above, the BBB is mainly composed of endothelial cells, which was reported to be responsible for 85% of the BBB 4, 7. Therefore, identifying new strategies to protect endothelial cells might be crucial for the treatment of BBB breakdown and TBI. Sphingolipids are important signaling molecules involved in many pathophysiological processes. S1P can play a role as an extracellular specific receptor ligand and an intracellular second messenger, but most of its role is mediated by its five homologous G-protein-coupled receptors, such as S1PR1. S1P and S1PRs cause different responses in different types of cells and tissues and it also remains unclear how will the S1P change after TBI. The S1P and S1PRs were reported to control a variety of physiological processes including vascular development and inflammation regulation 8, 24. Under normal physiological conditions, S1P and ceramide maintain a dynamic balance, in which S1P can bind to the S1PR1 receptor and play a beneficial role on endothelial cells 12, 25. We detected the expression of S1P after TBI, and the results showed that S1P began to rise 6 h after TBI, and the apoptosis of endothelial cells and the destruction of BBB were observed. The analogue of S1P is called FTY720. And FTY720 is the first FDA-approved oral S1PR1 modulator which is beneficial for multiple sclerosis 26. FY720 can internalize S1PR1 and continuously emit signals through adenosine cyclase inhibition and ERK phosphorylation, leading to cellular responses while S1P has no such effects 12, 25, 27. Our result showed a significant upregulation of S1PR1 following TBI at 24 h, so treatment targeting S1PR1 may have potential benefits for damaged BBB. We chose 24 hours as the node for the follow-up experiments. Our results suggested that FTY720 can reduce apoptosis of endothelial cells after TBI. In addition, our findings confirmed that the tight junction proteins were significantly elevated while S1PR1 was downregulated by FTY720 after TBI. We also tested the change of S1P after using FTY720, and the results showed that FTY720 had no effect on the content of S1P. This further demonstrates that the FTY720 and S1P are competitively combined with S1PR1. Besides, some recent studies have showed that S1P signaling is involved in the activation of ERK1/2 signaling 1. Lin et al. revealed that S1PR1 signaling activated ERK1/2 pathways in human pulmonary alveolar epithelial cells 28. A recent study suggested that in the TBI model, miR-873a-5p inhibited the transformation of microglia into M1 phenotype and reduced the inflammatory response by inhibiting phosphorylated ERK 29. Thus, we assessed whether FTY720 could modulate ERK1/2 pathway, which was proved to be important to endothelial cell apoptosis after TBI. Our results showed that activated (phosphorylated) ERK1/2 in the injured brain and the activated microglia were significantly increased after TBI and which was suppressed when treated with FTY720. This is consistent with the downstream effects of FTY720 mentioned earlier.

In the present study, we observed a significant difference in EB permeability assay between TBI + FTY720 group and TBI group. This finding showed that post-treatment with FTY720 stabilized the BBB after TBI. Claudin-5 is an important tight junction protein as mentioned above. Based on the findings of previous studies, it is revealed that more water transported through the BBB for the expression of claudin-5 was reduced after TBI, which lead to brain edema 30. In the present study, it was demonstrated that claudin-5 was elevated in the TBI + FTY720 group compared with the TBI + vehicle group, which is consistent with the result of brain water content analysis. Our findings indicated that FTY720 prevented brain edema by up-regulating claudin-5, which also suggest that claudin-5 may be a potential target for the treatment of TBI-related BBB breakdown.

In addition, astrocytes activation is an important feature in various CNS diseases 31. The activated astrocytes rapidly proliferate and migrate to the injured site to forming glial scar. It is generally believed that glial scar is a double-edged sword. In the acute phase of injury, activated astrocytes and their glial scar will limit the spread of lesions and reduce the inflammatory response of the damaged tissue 32. During the period of repairing, neovascularization and nerve regeneration occurs in the glial scar, but the axon elongation and synaptic formation are inhibited because of the scar, which inevitably has a negative impact 10, 24. Therefore, inhibiting the formation of the physical barrier of glial scar during convalescence is crucial to the prognosis after TBI. Besides, microglias are activated to clear damaged cells and tissues after trauma 33. However, normal tissues can also be damaged while activated microglia played the role of scavenger in acute phase of injury 34. As mentioned above, overactivated microglia can produce a large number of different types of inflammatory factors, which can cause neurotoxicity. For instance, TNF-α secreted by microglia induced cerebral immune and inflammatory responses, and then promoted the secondary brain injury 31. Furthermore, BBB dysfunction may cause circulating immune cells and many blood-derived substances to enter the brain, further increasing the activation of microglia and astrocytes, thereby enhancing the inflammatory response 35. These pathological processes lead to more neuron damage and edema formation. In the present study, we found that treatment with FTY720 reduced activation of astrocytes and microglia. Combined with the effects of FTY720 on BBB, it was suggested that FTY720 restored the structure of the NVU. Also, FTY720 can reduce inflammation in the brain after TBI by reducing the activation of microglia.

At last, it was found that there was significant difference on the mNSS between the sham and TBI groups on day 1, but no significant differences were identified from day 3 to 14 (Fig. 5D). On the first day, the score of the TBI + FTY720 group was about 4 points lower than the TBI group or the TBI + vehicle group. But on the next few days, the scores had no obvious differences between TBI + FTY720 and TBI + vehicle groups. This finding suggested that early application of FTY720 may improve neurological function in TBI mice 8, 24, which indicated the importance of early treatment. Survival analysis showed that there was significant difference in mortality between each group with TBI and the sham group (Fig. 5E). The final mortality was 20% in the FTY720 group, 65% in the TBI group and 45% in the vehicle group. This result suggested that FTY720 administration may increase the survival rate of TBI mice.

The present study focused on the status and function of endothelial cells after TBI. Inhibiting apoptosis of endothelial cells by FTY720 improved not only BBB function but also neurological function post-TBI. In addition, FTY720 attenuated the activation of astrocytes and microglia. All of these results suggested that FTY720 treatment leads to a better development of the NVU after TBI. The present study also proved that S1PR1 and related signaling pathways could be potential targets for the treatment of TBI.

## Supplementary Material

Supplementary figure.

## Abbreviations

TBI: traumatic brain injury
BBB: blood-brain barrier
NVU: neurovascular unit
SIPR: sphingosine-1-phosphate receptor
CNS: central nervous system
mNSS: modified neurological severity score
TUNEL: terminal deoxynucleotidyl transferase-mediated dUTP nick 3'-end labeling
