# Supplementary material for: FTY720 Reduces Endothelial Cell Apoptosis and Remodels Neurovascular Unit after Experimental Traumatic Brain Injury
Source: Int J Med Sci. 2021 Jan 1;18(2):304–13. doi: 10.7150/ijms.49066 (PMC7757143; doi:10.7150/ijms.49066)
Supplement: Supplementary file 1 — Supplementary figure. [file ijmsv18p0304s1.pdf]

FTY720 Reduces Endothelial Cell Apoptosis and Remodels Neurovascular Unit after Experimental Traumatic Brain Injury

Cheng et al.

Assay for S1P levels

ELISA tests were performed using a commercial kit (Echelon Biosciences, UT, USA). The grouping is shown above, S1P quantitative analysis was performed on all grouped brain tissue homogenate samples in duplicate. The levels were expressed as pmol/mg of sphingolipid.

Supplementary Figure

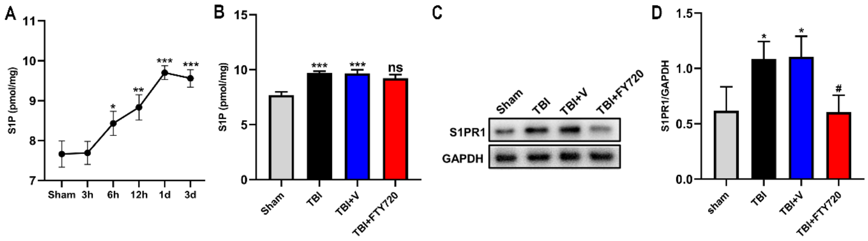

Supplementary Figure

A: The concentration of S1P in brain samples at various time points after TBI were measured by Elisa (n = 6 per group). Data were represented as the means ± SD (n = 6 per group), *p < 0.05, **p < 0.01, ***p < 0.001 versus the sham group.
B: FTY720 treatment had no effect on the content of S1P. Data are presented as mean ± SD (n = 6 per

group), *p < 0.05, **p < 0.01, ***p < 0.001 versus the sham group.

C-D: Protein levels of S1PR1 were measured by Western blot analysis. The content of S1PR1 was significantly reduced in the TBI + FTY720 group. Data are presented as mean ± SD (n = 6 per group), *p < 0.05, **p < 0.01, ***p < 0.001 versus the sham group; #p < 0.05, ##p < 0.01, ###p < 0.001 versus the TBI + vehicle group.
